# Acceleration of bone regeneration by activating Wnt/β-catenin signalling pathway via lithium released from lithium chloride/calcium phosphate cement in osteoporosis

**DOI:** 10.1038/srep45204

**Published:** 2017-03-24

**Authors:** Li Li, Xiaozhong Peng, Yongbao Qin, Renchong Wang, Jingli Tang, Xu Cui, Ting Wang, Wenlong Liu, Haobo Pan, Bing Li

**Affiliations:** 1 Department of Orthopedics, Fourth Affiliated Hospital of Guangxi Medical University/Liu Zhou Worker’s Hospital, Liuzhou, Guangxi 545005, China; 2 Center for Human Tissues and Organs Degeneration, Shenzhen Institutes of Advanced Technology, Chinese Academy of Sciences, Shenzhen, Guangdong 518055, China

## Abstract

By virtue of its excellent bioactivity and osteoconductivity, calcium phosphate cement (CPC) has been applied extensively in bone engineering. Doping a trace element into CPC can change physical characteristics and enhance osteogenesis. The trace element lithium has been demonstrated to stimulate the proliferation and differentiation of osteoblasts. We investigated the fracture-healing effect of osteoporotic defects with lithium-doped calcium phosphate cement (Li/CPC) and the underlying mechanism. Li/CPC bodies immersed in simulated body fluid converted gradually to hydroxyapatite. Li/CPC extracts stimulated the proliferation and differentiation of osteoblasts upon release of lithium ions (Li^+^) at 25.35 ± 0.12 to 50.74 ± 0.13 mg/l through activation of the Wnt/β-catenin pathway *in vitro*. We also examined the effect of locally administered Li^+^ on defects in rat tibia between CPC and Li/CPC *in vivo*. Micro-computed tomography and histological staining showed that Li/CPC had better osteogenesis by increasing bone mass and promoting repair in defects compared with CPC (P < 0.05). Li/CPC also showed better osteoconductivity and osseointegration. These findings suggest that local release of Li^+^ from Li/CPC may accelerate bone regeneration from injury through activation of the Wnt/β-catenin pathway in osteoporosis.

Osteoporosis is a common disease characterised by severe loss of bone mass and sparse microarchitecture, which frequently leads to fragility fractures^1^,^2^. The World Health Organization estimated that in 2004 osteoporosis caused >8.9 million fractures worldwide, and was seen mostly in elderly individuals. The International Osteoporosis Foundation has estimated that ≈70 million people aged >50 years have osteoporosis, and that 687,000 fragility fractures of the hip have occurred in China. In the USA and Europe, ≈30% of post-menopausal women have osteoporosis. Worldwide, at least 15–30% of men and 40% of women will suffer from one or more fragility fractures in their lifetime. Osteoporosis is underdiagnosed and undertreated because most of the population resides in rural areas.

The balance between the formation and resorption of bone plays an important part in regulation of bone mass in osteoporosis^3^. The Wnt/β-catenin signalling pathway (hereafter referred to as the “Wnt/β-catenin pathway”) also has an essential role in the formation and regeneration of bone. Activation of the Wnt/β-catenin pathway is achieved through binding of Wnt to low-density lipoprotein receptor-related protein 5 and 6 co-receptors and the 7-transmembrane domain-spanning frizzled receptor^4^,^5^. Signals are generated through the proteins Disheveled, Axin, and Frat-1, which disrupt the protein complex and downregulate the activity of glycogen synthase kinase (GSK)-3β, resulting in hypophosphorylation of β-catenin. Then, stabilised β-catenin aggregates in the cytosol and translocates to the nucleus. The transcriptional coactivator subsequently interacts with T cell factor/lymphoid enhancer binding factor (TCF/LEF). TCF/LEF are transcription factors that can mediate the effects of Wnts on gene transcription to upregulate osteoblast proliferation^6^.

Lithium chloride can suppress the activity of GSK-3β, which is considered to be a crucial regulator in the Wnt/β-catenin pathway^7^. Lithium chloride can activate the Wnt/β-catenin pathway by suppressing formation of the Axin-adenomatous polyposis coli (APC)-GSK-3β complex and preventing β-catenin phosphorylation, which increases bone formation^8^,^9^. Activation of β-catenin in the Wnt/β-catenin pathway *via* lithium treatment in mice is associated with an increase in bone mass and improvement in fracture healing^10^. Furthermore, treatment with lithium chloride also decreases the tumour burden in the skeleton and suppresses the development of myeloma *in vivo*^11^. In addition, lithium has been approved for the treatment of bipolar illness for >50 years^12^,^13^,^14^.

Repair of segmental bone defects resulting from cancer, trauma, or metabolic disorders is a major challenge in orthopaedic medicine. Calcium phosphate cement (CPC) was discovered first in 1985 by Chow and Brown and, since then, it has been popular in bone-tissue engineering^15^. CPC is a mixture of powder and liquid at a certain ratio and can form an injectable and remodelled paste that facilitates surgery and corrects bone defects readily^16^,^17^,^18^. Initially, CPC can be manipulated to fit the shape of various bone defects and give mechanical support to the lesion. Then, implants convert gradually to hydroxyapatite (HA) and integrate with surrounding regenerated osseous tissue, which show excellent osteoconductivity^19^,^20^,^21^.

Pilot studies by our research team have shown that lithium-doped calcium phosphate cement (Li/CPC) can promote the proliferation and differentiation of osteoblasts *in vivo*, and enhance osteogenesis in defects in rats compared with CPC^22^,^23^,^24^. In the present study, we explored the underlying mechanism of the proliferation and differentiation of cells under stimulation by lithium released from Li/CPC, and observed the role of Li/CPC in the repair of osteoporotic bone defects. We wished to ascertain if Li/CPC activates the Wnt/β-catenin pathway to induce osteogenesis and to treat orthopaedic diseases associated with reduced bone mass (e.g., osteoporosis).

## Results

### Composition and morphology of Li/CPC

**Figure 1:**
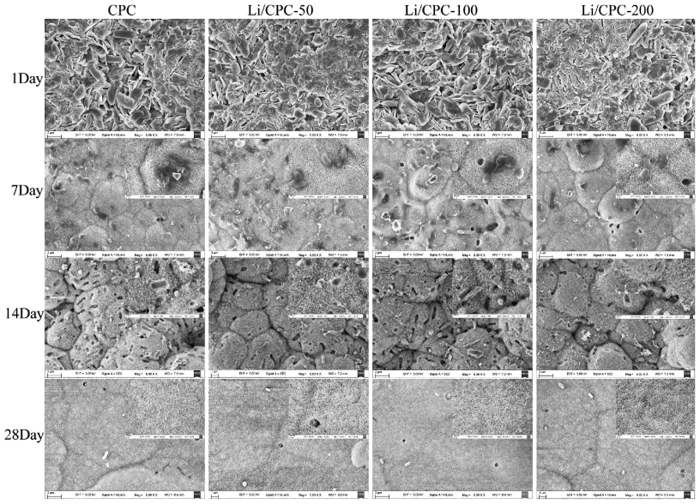
Characterisation of cement after immersion in simulated body fluid. SEM images indicated that original phases (TTCP, DCPA) had been converted gradually to flower-like or plate-like structures of HA at 1, 7, 14 and 28 days of immersion.

**Figure 2:**
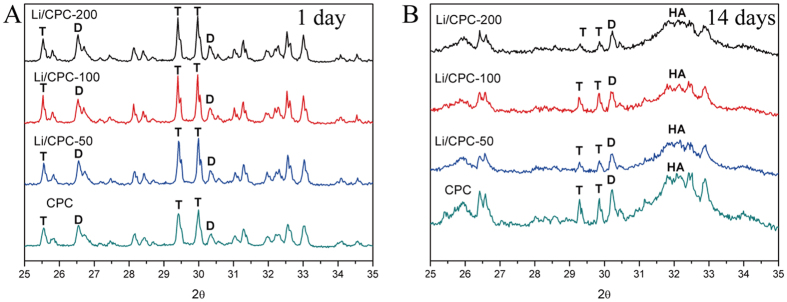
XRD analyses showed that the HA peak appeared at 1 and 14 days of immersion. (**A**) The original material peaks were present at 1 day, suggesting that cement composition was of initial materials (TTCP, DCPA). (**B**) A broad peak at ≈32° was observed at 14 days, suggesting HA formation.

At 1 day after immersion in simulated body fluid (SBF), the entangled structure of irregular crystals and irregular granular pattern were observed on the surfaces of cements under scanning electron microscopy (SEM), suggesting that the original materials were the major components of initial hardened cements. A typical flower-like structure was observed at 7 and 14 days, suggesting that HA with a poor crystalline structure had been formed gradually. Judging by the morphology of a plate-like structure at 28 days, the surface of cements was almost completely covered by precipitated HA (Fig. 1). In addition, X-ray diffraction (XRD) showed that the original material peaks were present and that the peak representing HA was absent after 1 day of immersion. This finding suggested that the composition of cements was initial materials [tetracalcium phosphate (TTCP), anhydrous dicalcium phosphate (DCPA)], and that HA did not form in the initial hardening of cements (Fig. 2A). After immersion in SBF for 14 days, the intensity of peaks of TTCP and DCPA decreased gradually. Simultaneously, a broad peak at ≈32° was observed, suggesting HA formation, which was consistent with the morphology of the cements under SEM (Fig. 2B). Combined with the results shown above, which consistently reflected HA formation, we concluded that Li/CPC had favourable bioactivity *in vitro* to the same extent as that of CPC, a finding that is in accordance with other reports^23^.

### Morphology of MC3T3-E1 cells

**Figure 3:**
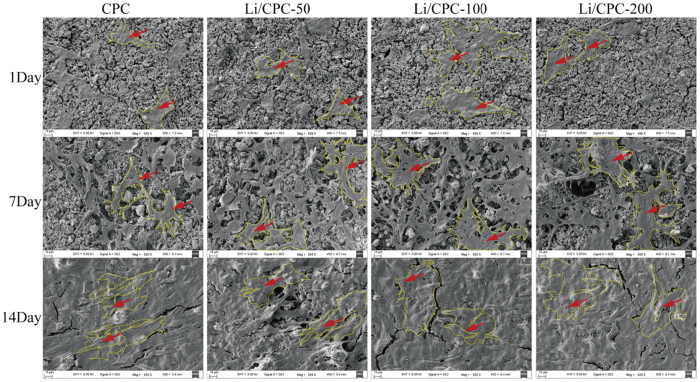
Morphology of MC3T3-E1 cells on the cement surface. With an increase in culture time, cements were covered by MC3T3-E1 cells gradually, indicating that cements had good biocompatibility. Red arrows pointed at MC3T3-E1 cells what were outlined by yellow line.

attaching to cements was examined using SEM. MC3T3-E1 cells were observed on the cement surface in all groups at 1 day. They showed well-spread and stretched filopodia to anchor to scaffold surfaces. At 7 days, cells spread to most scaffold surfaces, connected to adjacent cells or stretched numerous pseudopodia to anchor to scaffold surfaces. At 14 days, scaffold surfaces were covered completely with MC3T3-E1 cells in all groups and connected to each other *via* plasma extensions. This finding suggested that the cell-compatibility of all groups was satisfied (Fig. 3).

### Release of lithium ions (Li^+^) from cement

**Figure 4:**
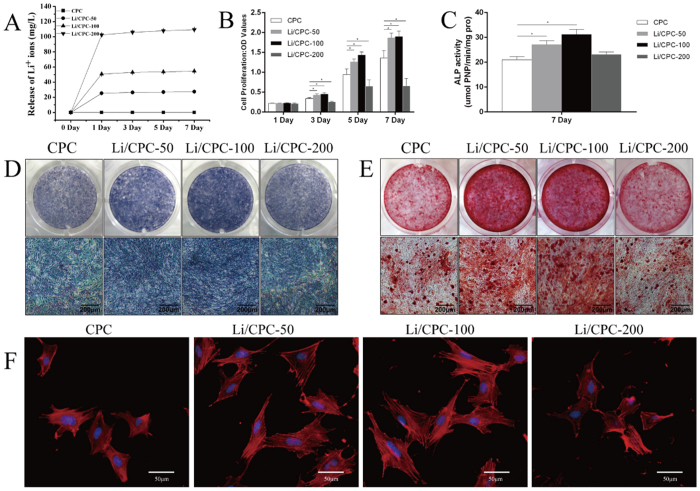
Effects of Li^+^ in extracts on proliferation and differentiation of MC3T3-E1 cells. (**A**) Li^+^ were released linearly from cements after immersion in medium for the same time, and most Li^+^ were released within 1day, only a small amount was released after 7 days (n = 5). (**B**) Cell proliferation on Li/CPC-50 and Li/CPC-100 was better at 3, 5 and 7 days than that on Li/CPC-200 and CPC (n = 5). (**C**) ALP activity at 7 days was better in Li/CPC-50 and Li/CPC-100 than for Li/CPC-200 and CPC (n = 5). (**D**,**E**) Osteogenic differentiation (ALP staining) and mineralisation (alizarin-red staining) of Li/CPC-50 and Li/CPC-100 were more obvious than those on Li/CPC-200 and CPC (n = 3). (**F**) Cells attached on Li/CPC-50 and Li/CPC-100 showed more spread and superior extension of filopodia, as well as greater focal adhesion *via* well-organized F-actin stress fibres (n = 3). Statistical analyses were done using Students’t-test. *p < 0.05 was considered significant.

Li^+^ released from cement with a higher concentration of lithium in Li/CPC was greater in extracts of materials at the same time. Most Li^+^ were released within 1 day and only a small amount was released after 7 days (Fig. 4A).

### Proliferation of MC3T3-E1 cells

At 1 day, cell number was maintained at the same level in all groups. Compared with CPC, the cell number in Li/CPC increased at 3, 5, and 7 days with Li^+^ release at 25.35 ± 0.12 to 50.74 ± 0.13 mg/l with culture time. However, a too-high concentration of Li^+^ (102.41 ± 0.11 mg/l) began to elicit cytotoxicity. Cell number in Li/CPC-200 was lower than that in Li/CPC-50 and Li/CPC-100, and the rate of cell proliferation in Li/CPC-200 was not significantly different compared with CPC (P > 0.05). The rate of cell proliferation on Li/CPC-100 was the highest of all groups tested (P < 0.05) (Fig. 4B).

### Differentiation of MC3T3-E1 cells

At 7 days, alkaline phosphatase (ALP) activity in Li/CPC was significantly higher than that in CPC. Differentiation on Li/CPC-100 was highest (P < 0.05) in all groups tested, and differentiation in Li/CPC-200 was not significantly different compared with CPC (P > 0.05) (Fig. 4C). Osteogenic differentiation and mineralisation of Li/CPC increased as Li^+^ were released at 25.35 ± 0.12 to 50.74 ± 0.13 mg/l, which reflected the calculation of ALP activity and staining with alizarin red (Fig. 4D,E).

### Staining of the cytoskeleton proteins of MC3T3-E1 cells

Figure 4F shows the cytoskeletons of MC3T3-E1 cells after immersion for 12 h in different extracts. Compared with CPC control, the attached cells for Li/CPC-50 and Li/CPC-100 extracts showed greater spreading and superior extension of filopodia, and greater focal adhesion *via* well-organised F-actin stress fibres (red filaments). However, the cytoskeletons of cells cultured with Li/CPC-200 were similar to those for CPC because the Li^+^ concentration increased excessively, and there was slight deterioration of MC3T3-E1 cells.

### Expression of osteogenic genes

**Figure 5:**
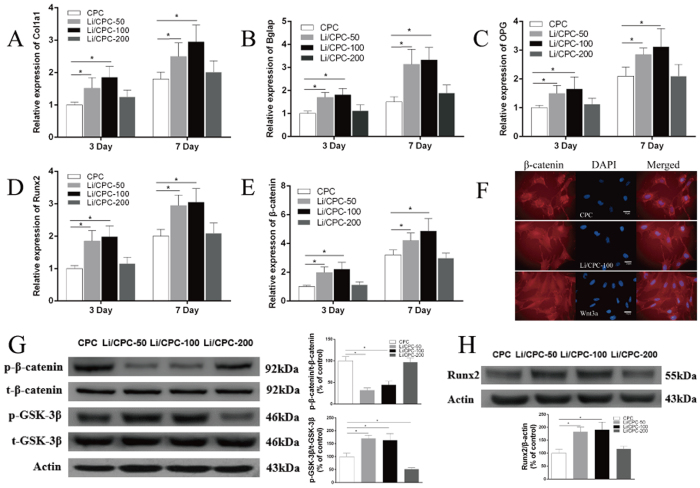
Activation of the Wnt/β-catenin signalling pathway *via* lithium released from Li/CPC. (**A**–**E**) Gene expression of Col1a1, Bglap, OPG, Runx2 and β-catenin were better in Li/CPC-50 and Li/CPC-100 than in CPC and Li/CPC-200 (n = 3). (**F**) β-catenin accumulation in the cytosol and translocation to the nucleus in Li/CPC-100 (n = 3). (**G**,**H**) Representative Western blot analysis of p-GSK-3β, t-GSK-3β, p-β-catenin, t-β-catenin and Runx2. Li/CPC-50 and Li/CPC-100 increased the amount of p-GSK-3β significantly and decreased the amount of p-β-catenin compared with Li/CPC-200 and CPC, expression of Runx2 was increased significantly in Li/CPC-50 and Li/CPC-100 compared with Li/CPC-200 and CPC. The band density was quantified using ImageJ software and data from three independent experiments were presented (CPC as the control group, the values were expressed as the mean ± SD, n = 3). Statistical analyses were done using Students’t-test. *p < 0.05 was considered significant.

Osteogenesis-related gene expression of osteoblastic markers [collagen type I alpha 1 (Col1a1), bone gamma-carboxyglutamate protein (Bglap), osteoprotegerin (OPG), runt-related transcription factor 2 (Runx2), β-catenin] were detected after incubation for 3 and 7 days with MC3T3-E1 cells in different extracts. In general, gene expression was time-dependent. After 3 days, expression of osteoblastic markers in the Li/CPC-50 and Li/CPC-100 was higher than that for CPC (P < 0.05), but there was no significant difference compared with Li/CPC-200 (P 0.05). Expression of osteoblastic markers was higher in Li/CPC-50 and Li/CPC-100 than that in CPC except for Li/CPC-200 at 7 days (P < 0.05) (Fig. 5A–E).

### Wnt/β-catenin pathway

We examined the effect of Li+ released from Li/CPC on activation of Wnt/β-catenin pathway. As the amount of phosphorylated GSK-3β increased, the amount of phosphorylated β-catenin decreased. This action stabilised β-catenin aggregated in the cytoplasm, which translocated to the nucleus when Li^+^ release was 25.35 ± 0.12 to 50.74 ± 0.13 mg/l (Fig. 5F,G). Li/CPC increased Runx2 expression significantly (P < 0.05) (Fig. 5H). Runx2 is essential for osteogenic differentiation as a Wnt/β-catenin/TCF target gene product. This result was similar to the result of *Runx2* gene expression.

### Rat model of osteoporosis

**Figure 6:**
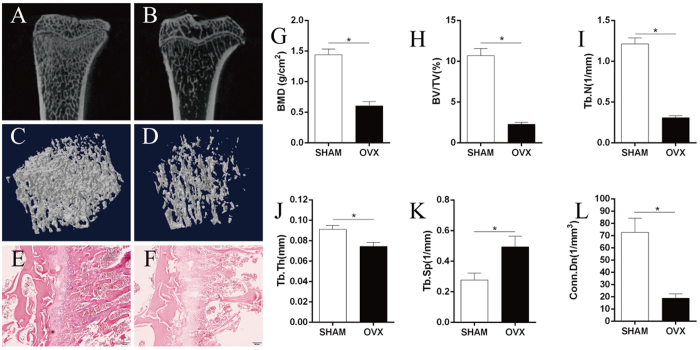
Establishment of a model of osteoporosis rats. (**A**–**D**) Micro-CT showed that OVX-treated rats had significantly less trabecular bone formation compared with the sham-operated group (n = 6). (**E**,**F**) Compared with the sham group, H&E staining of the tibia 3 months post-OVX showed significantly sparser trabecular bone in OVX rats (n = 6). (**G**–**K**) At 3 months after surgery, BMD, BV/TV, Tb.N, Tb.Th and Conn.D decreased relative to sham rats, and Tb.Sp increased (n = 6). Statistical analyses were done using Students’t-test. *p < 0.05 was considered significant.

Histomorphometric parameters and micro-architectural properties of bone were analysed 3 months after surgery *via* micro-computed tomography (CT) and histological staining of tibia-tissue sections. Bone mineral density (BMD), relative bone volume (BV/TV), trabecular number (Tb.N), trabecular thickness (Tb.Th) and connectivity density (Conn.D) decreased 3 months after surgery relative to sham rats, whereas trabecular separation (Tb.Sp) increased (P < 0.05). Three-dimensional (3D) micro-CT showed that rats that had undergone ovariectomy (OVX) had significantly less trabecular bone compared with sham-operated rats. Compared with the sham-operated group, histology images of tibias at 3 months post-OVX showed significantly sparser bone trabecular in OVX rats, which was consistent with 3D micro-CT results (Fig. 6).

### Micro-CT

**Figure 7:**
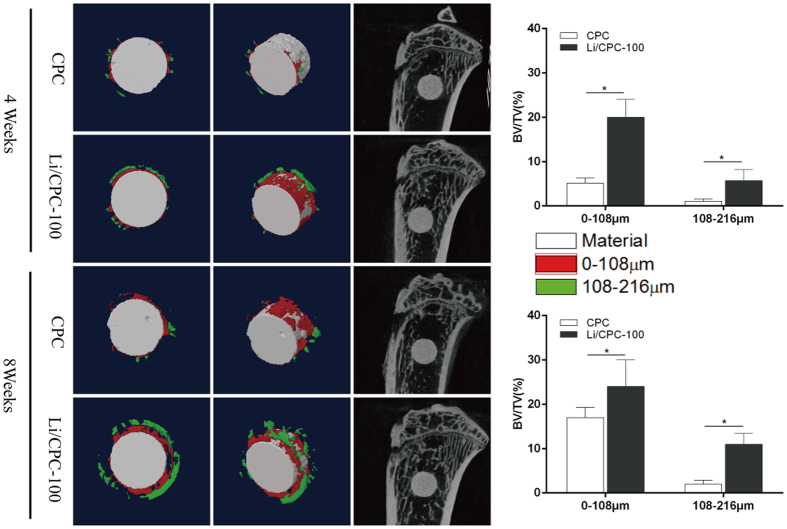
Micro-CT of rat tibial defects implanted with cements. Micro-CT showed the capacity of bone regeneration at varying distances from the periphery of implant was better in Li/CPC-100 than in CPC (n = 6). Statistical analyses were done using Students’t-test. *p < 0.05 was considered significant.

Bone defects were created by implantation of cylindrical material; such implantation was done in OVX rats. Micro-CT images and 3D computer models of tibial defects upon implantation were used to evaluate regenerated bone mass. At 4 weeks, a small amount of regenerated osseous tissue was found, and more extensive, newly formed bone occurred at 8 weeks (Fig. 7). Increased formation of new bone was detected around the Li/CPC compared with CPC at 4 and 8 weeks. The BV/TV at different distances from the surface of Li/CPC was significantly higher than that for CPC at 4 and 8 weeks (P < 0.05), which suggested that Li/CPC had better capacity for bone regeneration at interfacial areas.

### Histological staining

**Figure 8:**
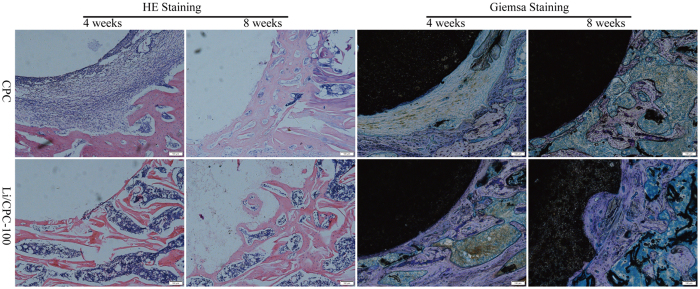
Histological staining. Images of H&E staining and Giemsa staining showed better *in vivo* osteoconductivity and osseointegration in Li/CPC-100 than in CPC at 4 and 8 weeks (n = 3).

OVX rats that had undergone filling of bone defects were killed. Specimens of the proximal tibia were collected for staining [haemotoxylin and eosin (H&E), Giemsa]. At 4 weeks, fibrous tissue was seen to infiltrate into a small gap between cement and bone in CPC. However, the initial gap was occupied entirely by new bone in Li/CPC. At 8 weeks, regenerated osseous tissue anchored to the surface of the implant was found in CPC and Li/CPC groups. However, this regenerated osseous tissue penetrated into the initial outer surface around Li/CPC, which suggested that Li/CPC could accelerate bone regeneration (Fig. 8).

## Discussion

CPC has been used for synthetic bone grafts in bone engineering because of its excellent bioactivity and osteoconductivity^25^,^26^,^27^,^28^. Li/CPC was manufactured by doping lithium chloride onto CPC. After immersion, testing of the hardened cement body was done to investigate bioactivity *in vitro*. With an increase in immersion time, cement morphology was changed gradually to the typical morphology seen in HA. This change was in accordance with the XRD-peak characteristics of HA, suggesting formation of a poorly crystalline apatite. HA has good biocompatibility and bioactivity^29^. Its chemical composition and crystalline structures are similar to those of apatite in human bone. Also, the molar ratio of calcium: phosphorus in HA is 1.67, which is close to that of human bone^30^. An exchange reaction of calcium ions in HA can occur with the carboxyl group present in amino acids, proteins, and organic acids, and new bone can be combined with implant material to provide a good growth interface for bone cells^31^,^32^. In addition, MC3T3-E1 cells exposed to cements also showed good biocompatibility at 14 days.

The Wnt/β-catenin pathway has an essential role in regeneration of osseous tissue by stimulation of the proliferation and differentiation of osteoblasts^33^,^34^. If signalling is blocked, phosphorylation of β-catenin is induced by a protein complex consisting of axin, adenomatous polyposis coli, and GSK-3β6. Lithium chloride can activate the Wnt/β-catenin pathway by inhibition of GSK-3β activity to stimulate the proliferation and differentiation of osteoblasts^35^.

The present study showed linear release of Li^+^ in the medium. That is, the lithium in Li/CPC is directly proportional to Li^+^ in the medium. The cell proliferation seen in Li/CPC-50 and Li/CPC-100 was better than that in Li/CPC-200 and CPC, which was in accordance with the results of cell-cytoskeleton staining. Early osteogenic differentiation (as reflected by ALP level) in Li/CPC-50 and Li/CPC-100 was better than that for Li/CPC-200 and CPC. As a marker of osteogenic differentiation, high expression of ALP suggests that a certain concentration of lithium doped to CPC favoured osteoblastic differentiation. Alizarin-red staining showed more calcium nodules, suggesting that a lithium concentration of 50–100 mM doped to CPC also favours osteoblastic mineralisation.

**Figure 9:**
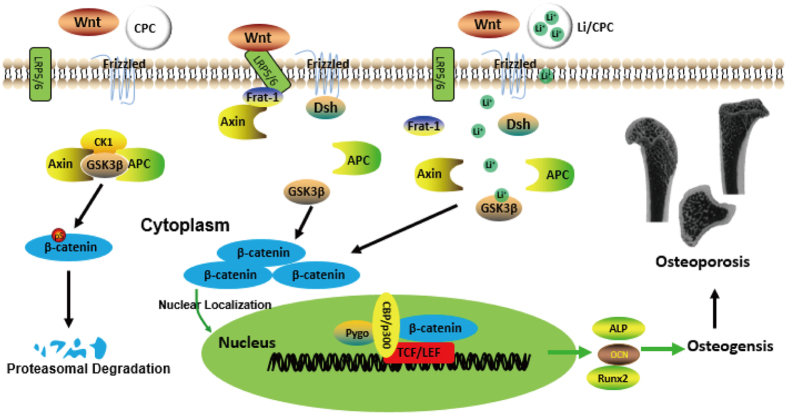
Mechanism of activation of the Wnt/β-catenin signalling pathway *via* lithium released from Li/CPC.

Studies on the Wnt/β-catenin pathway demonstrated that Li/CPC-50 and Li/CPC-100 increased the amount of phosphorylated GSK-3β significantly and decreased the amount of phosphorylated β-catenin. Then, stabilised β-catenin aggregated in the cytoplasm and translocated to the nucleus compared with Li/CPC-200 and CPC^36^. Reverse transcription-polymerase chain reaction (RT-PCR) showed that expression of osteogenesis-related genes was upregulated significantly in Li/CPC-50 and Li/CPC-100 compared with Li/CPC-200 and CPC, data that are in accordance with expression of the Wnt/β-catenin/TCF target gene product Runx2. These findings strongly suggest that the positive effect of Li^+^ release from Li/CPC at 25.35 ± 0.12 to 50.74 ± 0.13 mg/l on the proliferation, differentiation and mineralisation of MC3T3-E1 cells is associated with activation of the Wnt/β-catenin pathway (Fig. 9).

Osteoporosis is a systemic metabolic disease characterised by reduction of bone density, which leads to osteoporotic fractures. In osteoporosis, bone-formation capacity is weakened or bone-resorption capacity is enhanced relatively^37^,^38^. CPC has been used for treatment of non-load-bearing bone defects by virtue of its excellent bioactivity, biocompatibility and osteoconductivity, but it does not have special advantages for osteoporosis treatment. A GSK-3β inhibitor can activate the Wnt/β-catenin pathway to improve the microenvironment of bone cells^7^,^9^,^39^. Lithium had been used widely for the treatment of mental disorders (e.g., depression, mania) but increased bone mass and decreased bone transformation has been observed in these patients^40^,^41^. Hence, lithium is considered a candidate drug for osteoporosis treatment.

Li/CPC improved the proliferation, differentiation and biomineralisation of MC3T3-E1 cells. This action is achieved by stimulation of specific cellular responses at the molecular level upon Li^+^ release. In our study, female Sprague–Dawley rats underwent bilateral OVX. This osteoporotic rat model was established at 3 months post-OVX, which is in accordance with previous reports^42^,^43^. Micro-CT showed that Li/CPC enhanced osteogenesis significantly and elicited greater bone formation with higher BV/TV at the periphery of the implant material compared with CPC, indicating superior stimulation of osteogenesis *in vivo*. Early endochondral ossification and formation of new bone at 4 weeks, and penetration of regenerated osseous tissue into the initial outer surface at 8 weeks postoperatively in Li/CPC, suggested better osteoconductivity and osseointegration. We studied the effect of lithium-doped and undoped CPC on local osteogenesis. A sham-operated control group was not created and studied. Thus, we did not know the effect of bone fracture treated with or without implanted materials.

## Conclusions

The present study suggests that local application of Li/CPC to osteoporotic fractures can accelerate bone regeneration by activation of the Wnt/β-catenin pathway *via* lithium released from bioactive materials. Li/CPC maintained the bioactivity and biocompatibility of CPC, but also showed better osteoconductivity and osseointegration than CPC. Hence, Li/CPC may be a novel biomedical filling material for treatment of orthopaedic diseases with reduced bone volume, such as osteoporosis.

## Materials and Methods

### Preparation of Li/CPC

Li/CPC was prepared according to a method described previously^23^. TTCP (Sigma–Aldrich, Saint Louis, MO, USA) and DCPA (Sigma–Aldrich) were mixed at a ratio of 1:1 as a powder phase. Lithium chloride (0, 50, 100 and 200 mM; Sigma–Aldrich) was added to 20% wt of citric acid as a liquid phase (pH 4). Powder and liquid phases were mixed at a ratio of 0.3 ml/g and placed in a cylindrical mould to prepare the cement paste. A diameter of 12 mm and a height of 2 mm were used for material and cell-culture experiments, whereas 2.5 mm and 4 mm, respectively, were employed for animal studies.

### Composition and morphology

Cements were immersed in simulated body fluid (142 mM Na^+^, 5.0 mM K^+^, 1.5 mM Mg^2+^, 2.5 mM Ca^2+^, 147.8 mM Cl^−^, 4.2 mM HCO_3_^−^, 1.0 mM HPO_4_^2−^, 0.5 mM SO_4_^2−^, pH 7.4) at 37 °C. Then, 1/10 of the apparent surface area of the sample (mm^2^) was used to calculate the soak volume (ml), and liquid was changed every other day. The setting reaction was stopped by dipping in liquid nitrogen for 30 min after 1, 7, 14 and 28 days of immersion. Then, cements were stored at −80 °C and dried with a freeze-drying machine (Alpha 2-4LD plus; Martin Christ, Osterode am Harz, Germany). Morphology of the cement surface was observed under SEM (SUPRA 55; Zeiss, Oberkochen, Germany). Some cements were ground into powders and used for detection of the constitution and structure of the material phase by XRD (D8 Advance; Bruker, Billerica, MA, USA) using Cu Kα (γ = 1.5406 Å) radiation in step-scan mode (2θ = 0.02° per step).

### Preparation of extracts and differentiated solutions

Liquid extracts were prepared by immersion of cements in Dulbecco’s modified Eagle’s medium-alpha (DMEM-α; Hyclone, San Angelo, TX, USA) containing 10% FBS (Corning, Corning, NY, USA) and antibiotics (10 U/ml penicillin, 100 mg/ml streptomycin; Sigma–Aldrich) (1.25 cm^2^/ml, 37 °C, 24 h) according to International Standard Organization 10993-5 and the literature^23^. Differentiated solutions were prepared by supplementation with 100 nM dexamethasone (Sigma–Aldrich), 10 mM β-glycerophosphate (Sigma–Aldrich) and 0.2 mM ascorbic acid (Sigma–Aldrich) in different extracts.

### Li^+^ release from cements

Liquid extracts were prepared according to the methods described above. Li^+^ concentrations in cell culture media at 1, 3, 5 and 7 days were determined by inductively coupled plasma–atomic emission spectroscopy (JY2000-2; Horiba Jobin Yvon, Kyoto, Japan).

### Cell morphology

MC3T3-E1 cells (2 × 10^4^) were inoculated on cements placed in 24-well plates and cultured in DMEM-α containing 10% FBS. Cements were removed from wells after culture for 1, 7 and 14 days, washed with phosphate-buffered saline (PBS), and fixed in 4% paraformaldehyde for 1 h. Cement–cell constructs were dehydrated in a graded series of ethanol solutions (50, 70, 90, 95 and 100%) and dried with hexamethyldisilazane. Morphology of attached cells was observed using SEM (SUPRA 55; Zeiss).

### Cell proliferation

MC3T3-E1 cells (2 × 10^4^) were inoculated in 96-well plates (37 °C, 100% relative humidity, 5% CO_2_). Extracts were added to each well to replace the culture medium after 24 h. Cell proliferation was determined using cell counting kit-8 (Dojindo, Tokyo, Japan) at 1, 3, 5 and 7 days. The optical density (OD) of supernatants was detected by a spectrophotometer (Multiskan™ GO; Thermo Scientific, Waltham, MA, USA) at 450 nm.

### Assay to measure ALP activity

MC3T3-E1 cells (2 × 10^4^) were added to 24-well plates. After culture in cell-differentiated solutions for 7 days, supernatants were removed gently and the plates washed carefully with PBS. Cells on plates were homogenised in 0.2% Triton X-100 (200 μl). ALP activity was tested using an ALP Assay kit (Beyotime, Jiangsu, China). *p*-nitrophenol production was detected by monitoring OD using a microplate reader (Multiskan™ GO; Thermo Scientific) at 405 nm. The obtained value of OD was compared with the value from a standard curve of a series of diluted concentrations of *p*-nitrophenol in lysis buffer. The result of ALP activity was normalised by total protein content, which was measured with a Bicinchoninic Acid (BCA) Protein Assay kit (Thermo Scientific). The result was expressed as micromoles of *p*-nitrophenol formed per minute per microgram of total protein (1 μmol/min/mg protein).

### Staining to measure ALP activity

MC3T3-E1 cells (4 × 10^4^) were added to 24-well plates and cultured with cell-differentiated solutions for 14 days. ALP activity was determined using an ALP Enzymatic Activity Staining kit (Beyotime), which indicated ALP activity by the blue colour of the substrate (5-bromo-4-chloro-3-indolylphosphate/nitro-blue tetrazolium). Images were obtained under light microscopy (IX71; Olympus, Tokyo, Japan).

### Staining with alizarin red

MC3T3-E1 cells (1 × 10^5^) were added to six-well plates and cultured with cell-differentiated solutions for 21 days. The capacity for cell mineralisation was measured using Alizarin Red Staining kits (Sigma–Aldrich USA), which indicated cell mineralisation by changing the colour of the substrate to red. Images were obtained under light microscopy (IX71; Olympus).

### Staining of cytoskeleton proteins

MC3T3-E1 cells (2 × 10^4^) were added to glass coverslips in 24-well plates. After 24 h, extracts were added to each well to replace the culture medium for an additional 12 h. Then, cells were fixed with 4% paraformaldehyde for 10 min. Cells were permeabilised in permeabilisation buffer (0.2% Triton X-100) for 5 min. Then, coverslips were placed on a piece of Parafilm^®^ in a humid chamber and 200 μl of 100 nM rhodamine phalloidin (Cytoskeleton, Denver, CO, USA) added, followed by incubation in the dark for 30 min at room temperature. DNA was counterstained for 30 s with 200 μl of 4′,6-diamidino-2-phenylindole (DAPI; 100 nM; Dojindo) in PBS. Coverslips were rinsed in PBS and inverted on a drop of Antifade mounting media on a glass slide. Excess media were removed gently with a tissue and sealed on each side with nail polish. Images were taken with a fluorescence microscope (BX53; Olympus) to observe the cytoskeleton protein F-β-actin.

### Immunofluorescence staining

MC3T3-E1 cells (2 × 10^4^) were inoculated in glass coverslips. The culture medium, extracts of Li/CPC-100, and culture medium containing Wnt3a (R&D Systems, Minneapolis, MN, USA) were added to corresponding 24-well plate wells for 6 h. Cells were fixed in 4% paraformaldehyde for 15 min, and permeabilised in 0.2% Triton X-100 for 10 min. Cells were blocked with 1% bovine serum albumin in PBS for 1 h and incubated with monoclonal anti-β-catenin antibody (Abcam, Cambridge, UK) overnight at 4 °C. Subsequently, cells were incubated with anti-rabbit IgG (H + L; Invitrogen, Carlsbad, CA) for 1 h at room temperature. After washing in PBS and incubation in DAPI for 2 min, cell morphology was examined under a fluorescence microscope (BX53; Olympus).

### Gene expression by real-time RT-PCR

Expression of osteogenesis-related genes was evaluated using RT-PCR. MC3T3-E1 cells (5 × 10^4^) were added to 12-well plates, and cultured by cell-differentiated solutions for 3 and 7 days. Total RNA was isolated using TRIzol^®^ reagent (Invitrogen) and reverse transcription for mRNAs carried out using a Transcriptor First Strand cDNA Synthesis kit (Thermo Scientific) according to manufacturer instructions. Relative mRNA expression was determined using a SYBR Green qPCR kit (Toyobo, Osaka, Japan) and employing β-actin as the reference control. Expression of target mRNA was calculated from delta–delta Ct values. β-actin was used as an internal control. Primer sequences are listed in Table 1.

### Western blotting

Cultured MC3T3-E1 cells were harvested in lysis buffer (Beyotime) after different extracts had been supplemented with 100 nM dexamethasone, 0.2 mM ascorbic acid, and 10 mM β-glycerophosphate for 48 h. Protein concentration was measured using a BCA kit (Thermo Scientific). Total protein (80 μg) was separated on 8% polyacrylamide gel and transferred onto polyvinylidene difluoride membranes (Merck Millipore, Billerica, MA, USA). Membranes were blocked for 2 h at room temperature in 5% non-fat powdered milk in Tris-buffer, followed by incubation at 4 °C with primary antibodies to β-actin (Santa Cruz Biotechnology, Santa Cruz, CA, USA), β-catenin, p-β-catenin, Runx2 (Cell Signaling Technology, Danvers, MA, USA), GSK-3β, and p-GSK-3β (Abcam, UK). Bound primary antibodies were recognised by horseradish peroxidase-linked secondary antibodies (Santa Cruz Biotechnology). Protein bands were visualised using an enhanced chemiluminescence substrate kit (Millipore) and exposed to a ChemiDoc™ XRS chemiluminescence imaging system (Bio-Rad Laboratories, Hercules, CA, USA).

### Osteoporosis model in rats

Animal experiments were undertaken in accordance with guidelines outlined by the Shenzhen Institutes of Advanced Technology, Chinese Academy of Sciences (SIAT-IRB-160304-YYS-PHB-A0220) in Shenzhen, China, and approved by the Committee on the use of Live Animals in Teaching and Research of the same institute. Bilateral OVX was conducted on 6-month-old female Sprague–Dawley rats according methods described previously^43^. The BMD, BV/TV, Tb.N, Tb.Th, Tb.Sp and Conn.D of trabecular bone at proximal tibiae was analysed by micro-CT (1176; SkyScan, Kontich, Belgium) 3 months post-OVX.

### Material implantation

A second surgical procedure was carried out 3 months after establishment of the OVX model to create bone defects at the medial aspect of the tibial shaft, below the tibial plateau, bilaterally. Routine shaving and aseptic procedures were done. An incision was made to expose the tibia, creating a bone defect (diameter, 2.5 mm; depth, 4 mm) which was filled with Li/CPC-100 or CPC. The incision was closed with sutures. Animals were killed 4 and 8 weeks postoperatively.

### Micro-CT

Was done to evaluate the ultrastructure and morphology of defects. Raw images were reconstructed in 3D and converted to binary images with adaptive local thresholding. The new BV/TV at various distances from the cement surface (6 and 12 pixels; 1 pixel ≈18 μm) was calculated.

### H&E staining

Specimens of proximal tibia were fixed in 4% paraformaldehyde, decalcified in 10% ethylenediamine tetraacetic acid, and dehydrated in a graded series of ethanol solutions (70, 80, 90 and 100%). Specimens were embedded in paraffin and sectioned (thickness, 5 μm). H&E staining was used to observe regenerated osseous tissue and monitor specific tissue responses to implanted materials under light microscopy (BX53; Olympus). Five sections of each specimen were produced.

### Giemsa staining

Specimens were fixed in 4% paraformaldehyde and dehydrated in a graded series of ethanol solutions (70, 95, and 100%). Specimens were immersed in xylene and embedded in methyl methacrylate (Merck, Kenilworth, NJ, USA). Specimens were cut into sections (thickness ≈300 mm) by a hard tissue microtome (310 CP Band System; Exakt, Norderstedt, Germany). Five sections of each specimen were produced. Then, a micro grinding system (310 CP; Exakt) was applied to polish the sections down to a thickness of 30–40 mm. Giemsa staining (Merck) was used to observe regenerated osseous tissue under light microscopy (BX53; Olympus).

### Statistical analyses

Data are the mean ± SD of triplicate experiments. Statistical analyses were done using Students’*t*-test. P < 0.05 was considered significant. SPSS v21.0 (IBM, Armonk, NY, USA) was used in the study.

## Additional Information

**How to cite this article:** Li, L. *et al*. Acceleration of bone regeneration by activating Wnt/β-catenin signalling pathway via lithium released from lithium chloride/calcium phosphate cement in osteoporosis. *Sci. Rep.* **7**, 45204; doi: 10.1038/srep45204 (2017).

**Publisher's note:** Springer Nature remains neutral with regard to jurisdictional claims in published maps and institutional affiliations.

## Figures and Tables

**Table 1:** Primer sequences used for RT-PCR.

Name	Sequence	Gene bank
Col1a1	5′-GCTCCTCTTAGGGGCCACT-3′	NM_007742
	3′-CCACGTCTCACCATTGGGG-5′	
Bglap	5′-CTGACCTCACAGATCCCAAGC-3′	NM_007541.3
	3′-TGGTCTGATAGCTCGTCACAAG-5′	
OPG	5′-CAGCATCGCTCTGTTCCTGTA-3′	NM_011613.3
	3′-CTGCGTTTTCATGGAGTCTCA-5′	
Runx2	5′-AGAGTCAGATTACAGATCCAGG-3′	NM_001145920.2
	3′-TGGTCTTCTTACTGAGAGAGG-5′	
β-catenin	5′-ATGGAGCCGGACAGAAAAGC-3′	NM_007614.2
	3′-CTTGCCACTCAGGGAAGGA-5′	
β-actin	5′-GGCTGTATTCCCCTCCATCG-3′	NM_007393.5
	3′-CCAGTTGGTAACAATGCCATGT-5′	

Collagen type I alpha 1 (Col1a1), bone gamma-carboxyglutamate protein (Bglap), osteoprotegerin (OPG), runt-related transcription factor 2 (Runx2).
